# Fungi-Induced Upper and Lower Respiratory Tract Allergic Diseases: One Entity

**DOI:** 10.3389/fmicb.2018.00583

**Published:** 2018-04-03

**Authors:** Aleksandra Barac, David S. Y. Ong, Ljiljana Jovancevic, Aleksandar Peric, Pavol Surda, Vesna Tomic Spiric, Salvatore Rubino

**Affiliations:** ^1^Clinic for Infectious and Tropical Diseases, Clinical Centre of Serbia, Belgrade, Serbia; ^2^Faculty of Medicine, University of Belgrade, Belgrade, Serbia; ^3^Department of Medical Microbiology and Infection Prevention, Franciscus Gasthuis and Vlietland, Rotterdam, Netherlands; ^4^Department of Epidemiology, University Medical Centre Utrecht, Utrecht, Netherlands; ^5^Department of Otorhinolaryngology, Clinical Centre of Vojvodina, Novi Sad, Serbia; ^6^Faculty of Medicine, University of Novi Sad, Novi Sad, Serbia; ^7^Department of Otorhinolaryngology, Military Medical Academy, Belgrade, Serbia; ^8^Department of Otorhinolaryngology, Guy's and St Thomas' University Hospital, London, United Kingdom; ^9^Clinic for Allergology and Immunology, Clinical Centre of Serbia, Belgrade, Serbia; ^10^Department of Biomedical Sciences, University of Sassari, Sassari, Italy

**Keywords:** united airway *Aspergillus* disease, allergic bronchopulmonary aspergillosis, allergic *Aspergillus* sinusitis, asthma, chronic rhinosinusitis, respiratory tract

## Abstract

**Introduction:** *Aspergillus* can cause different allergic diseases including allergic bronchopulmonary aspergillosis (ABPA) and allergic fungal rhinosinusitis (AFRS). ABPA is allergic pulmonary disease against *Aspergillus* antigens. AFRS is a type of chronic rhinosinusitis (CRS) presented as hypersensitivity reactions to the fungal presence in sinuses. The aim of the present study was to clarify if ABPA and AFRS could be considered as a common disease entity.

**Methodology:** The prospective cohort study included 75 patients with ABPA. Patients were divided into two groups and compared with each other: (i) patients with CT confirmation of rhinosinusitis and presence of fungi in sinuses (ABPA+AFRS group) and (ii) patients without CT or without mycological evidence of AFRS (ABPA group).

**Results:** Findings of this study were: (i) AFRS was confirmed in 80% of patients with ABPA; (ii) all ABPA+AFRS patients had allergic mucin while fungal hyphae were present in 60% sinonasal aspirate; (iii) ABPA+AFRS patients had more often complicated CRS with (nasal polyps) NP (*p* < 0.001) and more severe forms of CRS; (iv) culture of sinonasal aspirate revealed fungal presence in 97% patients with ABPA+AFRS; (v) patients with ABPA+AFRS had more common positive skin prick test (SPT) for *A. fumigatus* (*p* = 0.037), while patients without AFRS had more common positive SPT for *Alternaria alternata* and *Penicillium notatum* (*p* = 0.04 and *p* = 0.03, respectively); (vi) 67% of ABPA patients had *Aspergillus* induced AFRS; (vii) larger number of fungi was isolated from the air-samples obtained from homes of patients with ABPA+AFRS than from the homes of patients without AFRS, while the most predominant species were *A. fumigatus* and *A. niger* isolated from almost 50% of the air-samples.

**Conclusion:** The pathogenesis of ABPA and AFRS is similar, and AFRS can be considered as the upper airway counterpart of ABPA. Fungi-induced upper and lower respiratory tract allergic diseases present common entity. Next studies should clarify the mechanism by which fungi turn from “normal flora” into trigger of immunological reactions, resulting in ABPA or AFRS as well as to find new approaches for its' diagnosis and treatment.

## Introduction

The respiratory tract is continuously exposed to fungal spores present in the environment, and studies showed viable fungus present in the high rates in sinonasal mucus and bronchial sputum cultures, even in healthy subjects (Ponikau et al., 1999; Buzina et al., 2003). Fungal presence is commonly considered as colonization, but it may be an important extrinsic trigger for upper and lower airway allergic diseases especially in patients with asthma, chronic rhinosinusitis (CRS), cystic fibrosis and allergic rhinitis (Kumamoto, 2016). Understanding of fungi-induced allergic airway diseases is complicated by the enormous biodiversity of the fungi, problems with defining major allergens, unclear pathogenesis, and the role of fungi as allergens. Microbial communities, especially fungi, interact with environment, and host inflammatory response could cause or mediate the inflammatory process of upper and lower airway disease (Huffnagle and Noverr, 2013).

One of the most common pathogenic fungi causing upper and lower airway disorders are *Aspergillus* species (Chowdhary et al., 2016; Rick et al., 2016). *Aspergillus* can be found throughout the world. Its spores are ubiquitous and present almost everywhere in the human environment, outdoor and indoor (Woolnough et al., 2015; Chowdhary et al., 2016). In a recent study with patients with CRS, fungal presence in sinuses was identified in 63% patients, while *Aspergillus* was the most predominant fungal genus (Zhao et al., 2017).

Given the ubiquitous nature of fungi, exposure is unavoidable, however, it is not yet known how fungi turn from colonization into triggers of inflammatory reactions, resulting in allergic bronchopulmonary aspergillosis (ABPA) and allergic fungal rhinosinusitis (AFRS) (Lackner et al., 2005). AFRS and ABPA have many similarities: increased levels of serum IgE, and *Aspergillus*-specific IgE and IgG antibodies (Ab), similar immunopathology, and treatment (Chakrabarti et al., 2009; Shin et al., 2015; Barac et al., 2018). Bronchial mucus present in ABPA patients and allergic mucin present in AFRS patients are histologically identical and in some cases contain fungal hyphae, while the culture reveal the presence of different fungal taxa, mostly *Aspergillus* sp. (Chakrabarti et al., 2009; Barac et al., 2015; Kale et al., 2015). Our hypothesis is that fungal infections of upper and lower respiratory tract represent one disease, “united fungal airway disease,” as they both depend on the same multiple factors such as exposure levels, anatomy, mucociliary clearance, mucosal health, and host immune factors (Ryan and Clark, 2015).

Literature review identified several review articles on related topic (Ryan and Clark, 2015; Kim, 2016; Rick et al., 2016; Agarwal et al., 2017) but there is a lack of comprehensive prospective study examining paired upper and lower airway mycobiota, clinical and other relevant findings in individuals with AFRS and ABPA. Based on these grounds, we performed a prospective cohort study in patients with upper and lower airway fungi-induced allergic diseases with the aim to further examine the “united airway fungal disease” hypothesis.

## Methodology

### Study population

This prospective cohort study was conducted at the Clinical Centre of Serbia, Faculty of Medicine, University of Belgrade, from 1st February to 1st December 2016. The study was approved by the Ethical Committee of Clinical Centre of Serbia (5030/5) and the Ethical committee of Faculty of Medicine University of Belgrade (29/VI-3). Informed consent has been obtained from all study patients.

### Inclusion criteria

The study included consecutive patients with ABPA presented to ENT department of our hospital. A diagnosis of ABPA was made when at least six of the primary diagnostic Rosenberg-Patterson criteria were fulfilled (Rosenberg et al., 1977; Ishiguro et al., 2016). Diagnoses of ABPA was established by consensus among clinicians, radiologists, and mycologists based on the diagnostic criteria, laboratory findings, or histologic findings obtained via bronchoscopic or thoracoscopic lung biopsy (Ishiguro et al., 2016).

Patients with already confirmed diagnosis of ABPA were included in the study if they fulfilled inclusion criteria: (i) >16 years; (ii) no treatment with systemic corticosteroids over last 7 days and local corticosteroids for 3 days before inclusion, and (iii) absence of invasive fungal infection (screened by serology testing of anti-*Aspergillus* and anti-*Candida* IgM and IgG Ab, as well as the concentration of galactomannan and mannan in patients' sera).

We aimed to reveal how many patients suffer with ABPA and concomitant AFRS. Therefore, once the diagnosis of ABPA was made, we performed further AFRS diagnostic workup including CT imaging and sinonasal aspirate analysis. We defined AFRS as a presence of the hyperattenuating signal density visualized by CT imaging (“double density” sign), presence of allergic mucus and fungi grew from the culture of sinonasal aspirate. Finally, patients were divided into two groups depending on the presence of AFRS: (i) group A (ABPA+AFRS patients) and (ii) group B (patients with only ABPA but without AFRS).

### Data collection

The following data included: (i) collection of patient's demographics and history data including number of previous endoscopic surgery of sinuses, duration of CRS, duration of AFRS, previous use of local corticosteroids during 3 months, presence of other co-morbidities (asthma, cystic fibrosis, allergic rhinitis, CRS); (ii) examination for fungal allergy: total sera IgE Ab, absolute eosinophil count in blood and skin prick test on fungal allergens (SPT); (iii) anterior rhinoscopy; (iv) CT imaging of paranasal sinuses and thorax; (v) microbiological analysis of induced aspirate; and (vi) cultivation of air samples from patients' homes (bedrooms).

### Total serum IgE, eosinophil count and SPT

A concentration of total IgE Ab in serum was measured by enzyme-linked immunosorbent assay (ELISA; Euroimmun AG, Germany). Results were interpreted as follows: (i) negative (<100 kU/L), (ii) low positive (100–500 kU/l) and (iii) high positive (≥500 kU/l). The blood sample was taken before SPT and put into the tube containing ethylene-diamine-tetra-acetic acid (EDTA). Eosinophil counting was performed with Fuchs-Rosenthal counting chamber. Results <350 mm^3^ were considered as negative. SPT was done for the most common fungal allergens: *A. fumigatus, Alternaria alternata*, and *Penicillium notatum*.

### CT imaging and anterior rhinoscopy

All patients underwent sinonasal and chest CT imaging. In addition, all patients were examined by ear, nose, and throat (ENT) specialist to confirm AFRS presence. The presence of nasal symptoms (nasal obstruction, nasal secretion, postnasal discharge, impaired or lost sense of smell, facial sense of pressure) for more than 12 weeks and nasal rhinoscopic/endoscopic finding including the presence of characteristic allergic mucin (thick and viscous, often brown and yellow in color) and unilateral or bilateral nasal polyps and soft-tissue opacification of nasal cavity/paranasal sinuses with the presence of serpiginous areas of high attenuation, suggest the diagnosis of AFRS.

### Mycological analyses

**Figure 1:**
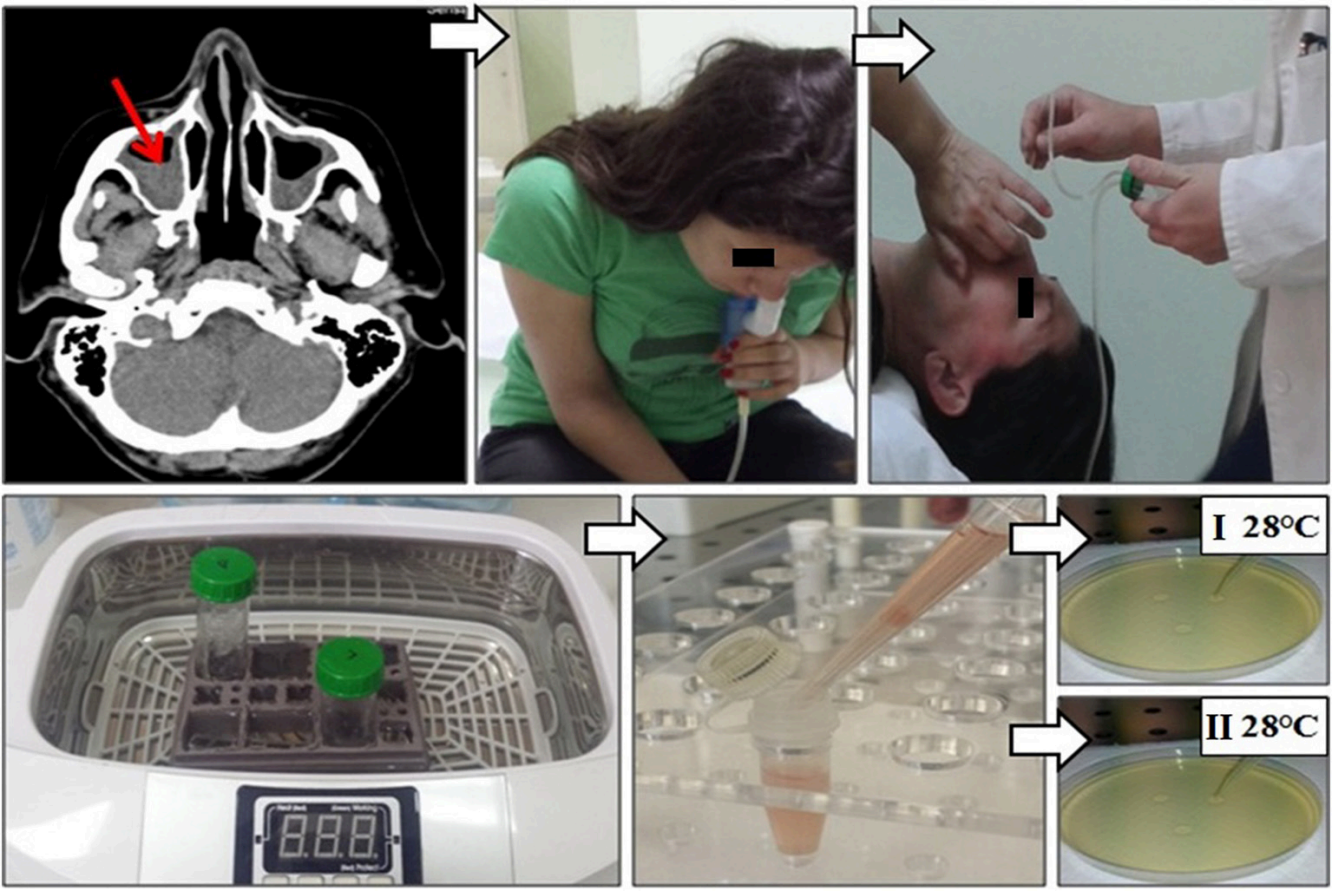
Sinonasal aspirate processing algorithm: (i) CT imaging of paranasal sinuses of patients with ABPA revealed CRS; (ii) each patient did the inhalation by PARI-SINUS nebulizer with hypertonic-NaCl solution for 10 min in the quadruped position; (iii) after the inhalation mucin was collected from the sinuses by aspiration with mucus extractor in a supine position with head tilted backward; (iv) the sinonasal aspirate was additionally processed in ultrasound cleaner aimed to prepare single-cell solution, and (v) afterwards single-cell solution was cultured at Sabouraud dextrose agar at 28°C for 7 days. Fungi were regarded as causative when the same fungi was isolated from sinonasal aspirate repeatedly.

Before sampling induced sinonasal aspirate, nasal cavities were pre-treated with cotton swabs aimed to decrease the contamination possibility. Afterward, each patient did the inhalation by PARI-SINUS nebulizer with 5 ml hypertonic-NaCl solution for 10 min in the quadruped position (PARI, Starnberg, Germany) (Figure 1). Quadruped position facilitates drainage of the content from paranasal sinuses (Ford et al., 2011). After the inhalation, mucin was collected from the sinuses by aspiration in a supine position with head tilted backward at a 30° angle. Aspiration was done with mucus extractor (ULTRAMED, Asyut, Egypt), and the obtained sample was additionally processed in ultrasound cleaner (BlueWave Ultrasonic, Davenport, IA, USA). The same sampling procedure has been repeated twice within 7 days. Sabouraud dextrose agar was used for fungal culturing; plates were incubated at 28°C for 7 days. Identification of fungi was based on macroscopic and microscopic characteristics using standard mycological methods (McClenny, 2005). Fungi were regarded as causative if the same fungus was isolated from induced sinonasal aspirate from the first and repeated sinonasal aspirate (Lebowitz et al., 2002; Yeo and Wong, 2002).

### Air sampling

**Figure 2:**
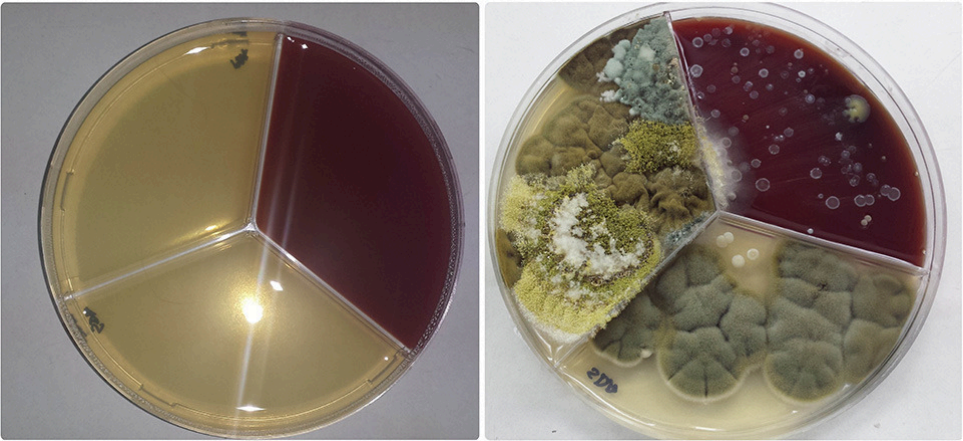
Air-sampling protocol: Petri dish with Potato dextrose agar has been left for 1 h at room temperature in patients bedrooms.

Patients were asked to leave open Petri dishes with potato dextrose agar for 1 h at room temperature in their bedrooms (Figure 2). Afterward, Petri dishes that contain air-samples were fixed with parafilm (Sigma-Aldrich, USA) and deposited at the laboratory for further analysis. Plates were incubated at 28°C for 7 days. Identification of fungi was based on macroscopic and microscopic characteristics using standard mycological methods (McClenny, 2005).

### Statistical analysis

Descriptive and inferential statistical analyses were used for evaluation of data using Statistical Package for Social Science (SPSS 17.0, Chicago, IL, USA). Data were expressed as mean ± standard deviation (SD) and counts or percentages, where appropriate. The distribution of the continuous variables was checked using the Kolmogorov–Smirnov test which did not show normal distribution. Thus, non-parametric testing was used. Mann–Whitney test was used for nonparametric data, and χ^2^ test was used for categorical variables. All differences were considered significant at *p* < 0.05.

## Results

### Clinical characteristics and sociodemographic data of the cohort

In total, 75 consecutive patients who signed the written consent and fulfilled the ABPA criteria were enrolled into the study. Mean age of 75 patients was 36 ± 11 (range, 16–50) with an almost equal sex distribution (M:F ratio = 1.1:1). In total, 77% had only asthma, while 23% had asthma and bronchiectasis. Chest CT revealed transient pulmonary infiltrates and central bronchiectasis in 17/75 (23%) patients. All patients have increased total IgE Ab, while 72% had positive SPT for *A. fumigatus*, and 34 and 28% had co-occurrence of positive SPT for *A. fumigatus* and *Alternaria alternata* or *A. fumigatus* and *Penicillium notatum*, respectively. In total, 28% patients were solely sensitized to *Alternaria* or *Penicillium*, and this group was classified as allergic bronchopulmonary mycosis (ABPM).

### Clinical characteristics and sociodemographic data of ABPA+AFRS patients (group A)

**Figure 3:**
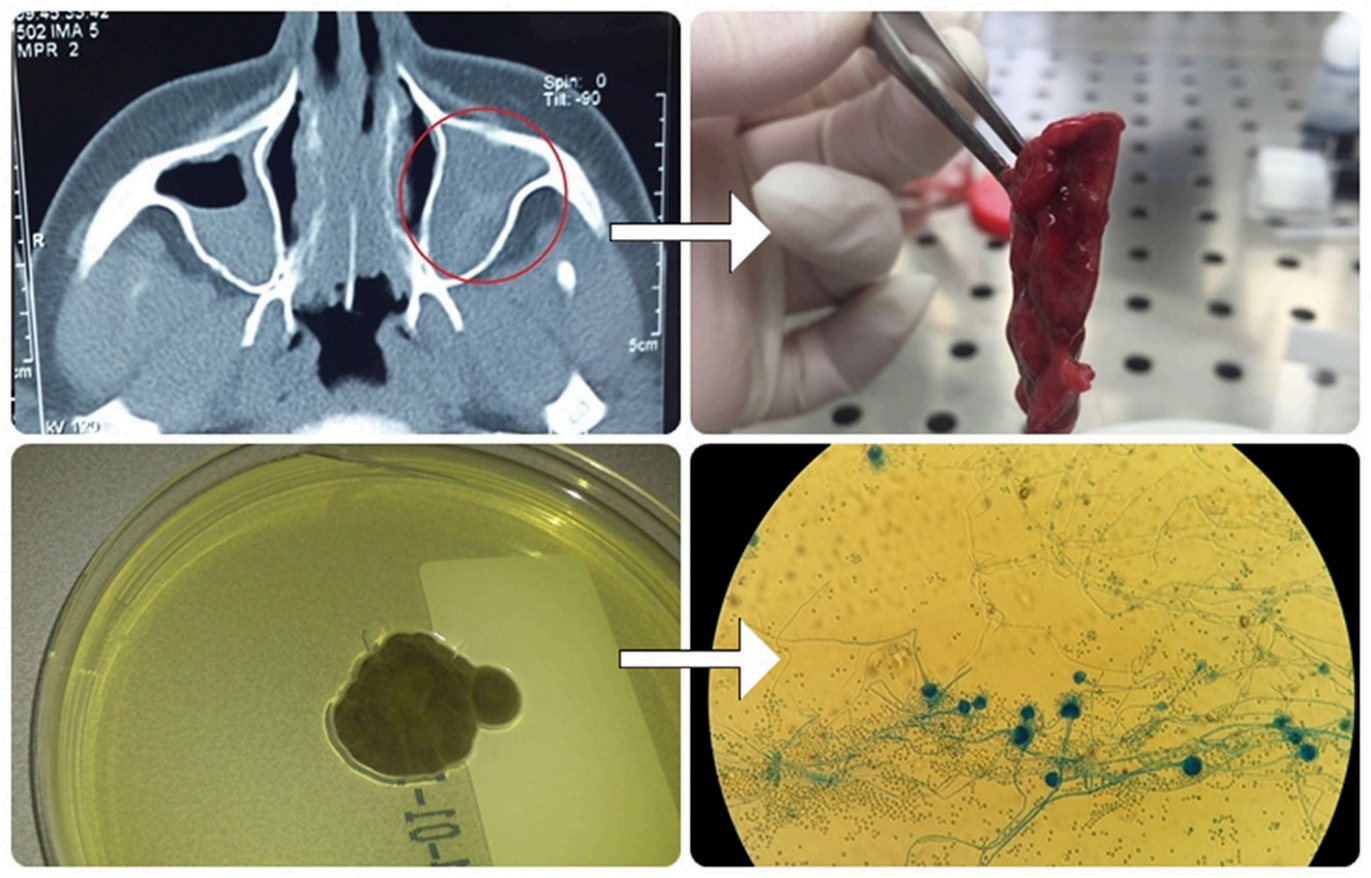
Algorithm for fungal detection in the sinuses: CT imaging of paranasal sinuses of patients with ABPA revealed AFRS; Aspiration of allergic mucin was done, processed and used for mycological examination by microscopy and culturing.

CRS was present in 82.7% (62/75) patients with ABPA. Mean duration of CRS was 12 ± 9 years (range 2–30 years). Complicated CRS with recalcitrant nasal polyposis (NP) and more than one previous endoscopic surgery of sinuses was found in 45.5% patients with ABPA. Rhinological score estimated by sinonasal outcome test (SNOT 22) for scoring of subjective symptoms revealed that 51.3% ABPA patients had severe symptoms CRS while CT imaging score revealed that 34.6% ABPA patients had severe CRS. In 60/75 (80%) ABPA patients CT of paranasal sinuses revealed mucosal thickening with hyperdense lesions, while fungi were isolated from sinonasal aspirate or allergic mucin from the sinuses of these patients, and they were characterized as ABPA+AFRS patients (group A). Mean age of patients from group B was 34 ± 9 (range 18–48) with predominance of male gender (M:F ratio = 1.6:1). Out of all ABPA+AFRS patients, 87% had only asthma, while 13% had asthma and bronchiectasis. Chest CT showed transient pulmonary infiltrates and central bronchiectasis in 9/60 (15%) patients. Sinonasal allergic mucin was seen in all ABPA+AFRS patients and fungal hyphae in 36/60 (60%) (Figure 3). All patients from group B had increased total IgE Ab and positive SPT for *A. fumigatus*, 14% had positive SPT for *Alternaria alternata* and 10% for *Penicillium notatum*.

### Clinical characteristics and sociodemographic data of patients with ABPA without AFRS (group B)

Patients with ABPA but without AFRS belong to group B. Out of all patients from group B, 48% had only asthma, while 52% had asthma and bronchiectasis. Other relevant sociodemographic and clinical data of this group are presented in Table 1.

**Table 1:** Relationship of sociodemographic data and clinical characteristics between patients with ABPA and AFRS and patients with ABPA only.

**Variables**	Group n (%)^**^			***p***
	**All patients *n* = 75 (100%)**	**Group A *n* = 60 (80%)**	**Group B *n* = 15 (20%)**	
**Sex**				^*^0.042
M	40 (53.3%)	37 (61.7%)	3 (20)	
**Age (mean** ± **SD)**				0.247
	36.12 ± 11.21	34.54 ± 9.14	37.18 ± 11.81	
**Chest CT**				^*^0.005
Pulmonary infiltrates and central bronchiectasis	17 (23.3)	9 (15.1)	8 (53.3)	
**Comorbidities**				^*^0.007
Asthma	58 (77.1)	52 (86.7)	7 (48)	
Asthma+bronchiectasis	17 (22.9)	8 (13.3)	8 (52)	
**CRS**				^*^ < 0.001
Yes	62 (82.7)	60 (100)	2 (13.3)	
**Duration of CRS (years)**				^*^ < 0.001
<5	18 (29.1)	16 (26.6)	2 (100)	
5–10	11 (17.7)	11 (18.4)	0 (0)	
10–20	9 (14.5)	9 (15)	0 (0)	
>20	24 (38.7)	24 (40)	0 (0)	
**Duration of CRS (years) (mean** ± **SD)**				0.427
	12.88 ± 9.5	14.24 ± 10.7	13.78 ± 9.81	
**CRS with NP**				^*^0.028
Yes	28 (37.3)	27 (45)	1 (6.7)	
**Rhinological index for objective assessment of CRS**				^*^ < 0.001
Mild	20 (32.3)	18 (29.5)	1 (50)	
Moderate	10 (16.4)	12 (20.5)	1 (50)	
Severe	32 (51.3)	30 (50)	0 (0)	
**CT index for objective assessment of CRS**				^*^ < 0.001
Mild	19 (31.5)	20 (33.8)	2 (100)	
Moderate	21 (33.9)	19 (32.8)	0 (0)	
Severe	22 (34.6)	21 (33.4)	0 (0)	
**Fungal hyphae**				^*^ < 0.001
Yes	36 (48)	36 (60)	0 (0)	
**SKIN PRICK TEST**				
*Aspergillus fumigatus*	54 (72.3)	60 (100)	4 (26.7)	^*^0.004
*Alternaria alternata*	26 (34.4)	9 (14.2)	7 (46.7)	^*^0.032
*Penicillium notatum*	21 (28.1)	6 (10.6)	5 (33.3)	^*^0.038

* *According to Chi-square test, p < 0.05; Abbreviations: CRS, chronic rhinosinusitis, CT, computerized tomography; NP, nasal polyposis; ABPA, allergic broncopulmonare aspergillosis*.

** *Group A (ABPA+AFRS patients); group B (patients with only ABPA but without AFRS)*.

### Relationship of clinical characteristics between group A and group B

AFRS was confirmed in a significant proportion (80%). There was a difference in male gender between group A and B (61.7% vs. 20%, respectively; *p* = 0.042). Patients from group B had more common bronchiectasis comparing to group A (53% vs. 15%, respectively; *p* = 0.001). Patients from group A had more often complicated CRS with NP (*p* < 0.001) and more severe forms of CRS with chronicity more than 10 years (*p* < 0.001), compared to group B. Allergic mucin was seen in all patients from group A, while it was not present in group B. Fungal hyphae in allergic mucin were seen in 60% of patients with AFRS. The culture of sinonasal aspirate revealed fungal presence in 97% patients with ABPA+AFRS, while there was no positive fungal growth in the sinonasal aspirate from the patients in group B.

All patients from both groups had increased total IgE Ab. Patients from group A had more common positive SPT for *A. fumigatus* (*p* = 0.037), while patients from group B had more common positive SPT for *Alternaria alternata* and *Penicillium notatum* (*p* = 0.04 and *p* = 0.032, respectively).

### Distribution of fungi isolated from sinonasal aspirate of ABPA+AFRS patients

**Figure 4:**
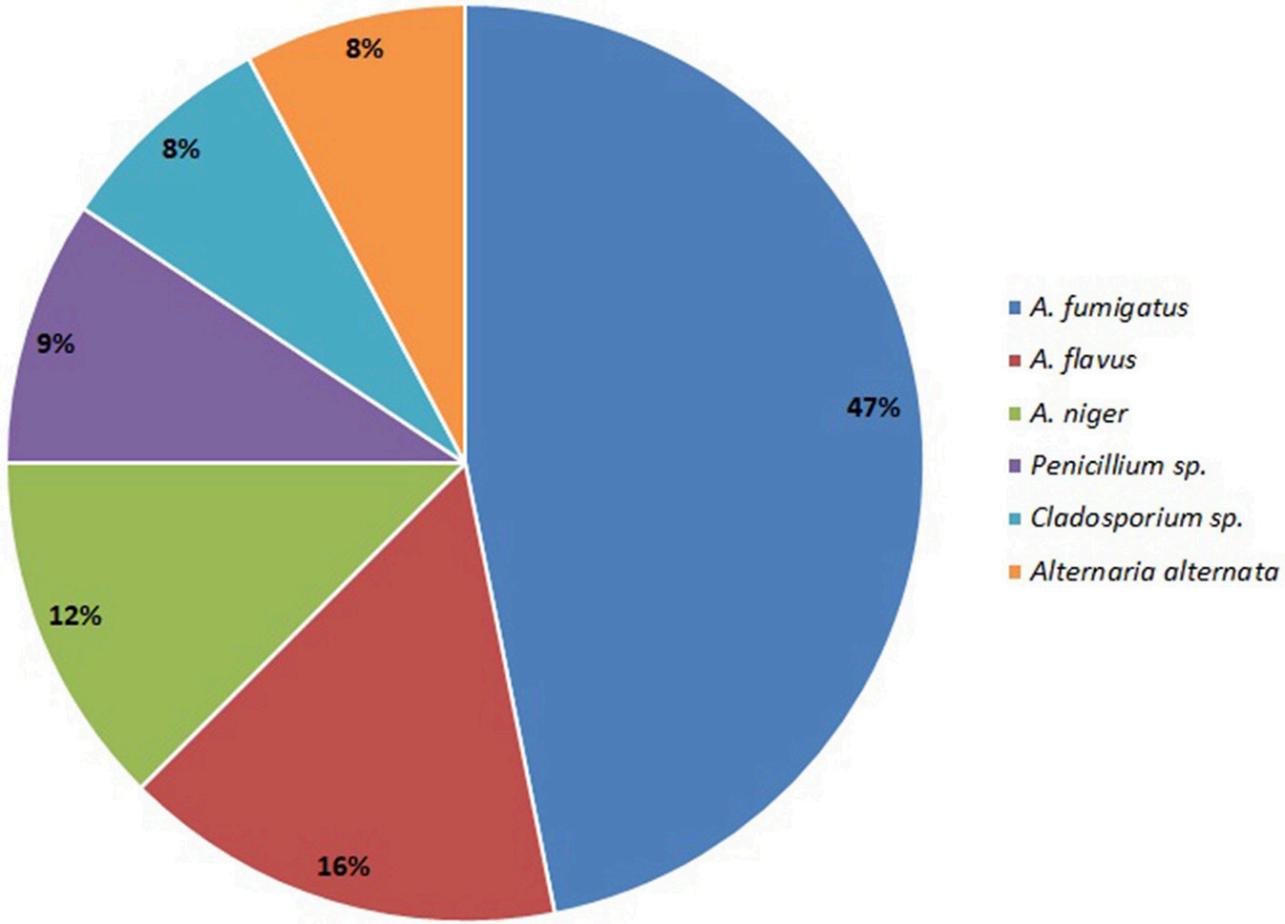
Distribution of fungal species in sinonasal aspirate of patients from patients AFRS+AFRS.

*Aspergillus* sp. was the most common isolate from sinonasal aspirate found in 80% patients with AFRS, and this group is classified as *Aspergillus* induced AFRS. *A. fumigatus* was isolated in 50% (30/60) cases, followed by *A. flavus* in 17% cases (10/60); *A. niger* in 13% (8/60) cases; *Penicillium* sp. (10%; 6/60); *Cladosporium* sp. (5%; 5/60) and *Alternaria alternata* (5%; 5/60) (Figure 4).

### Biodiversity of fungi in air samples obtained from homes of patients with ABPA

**Figure 5:**
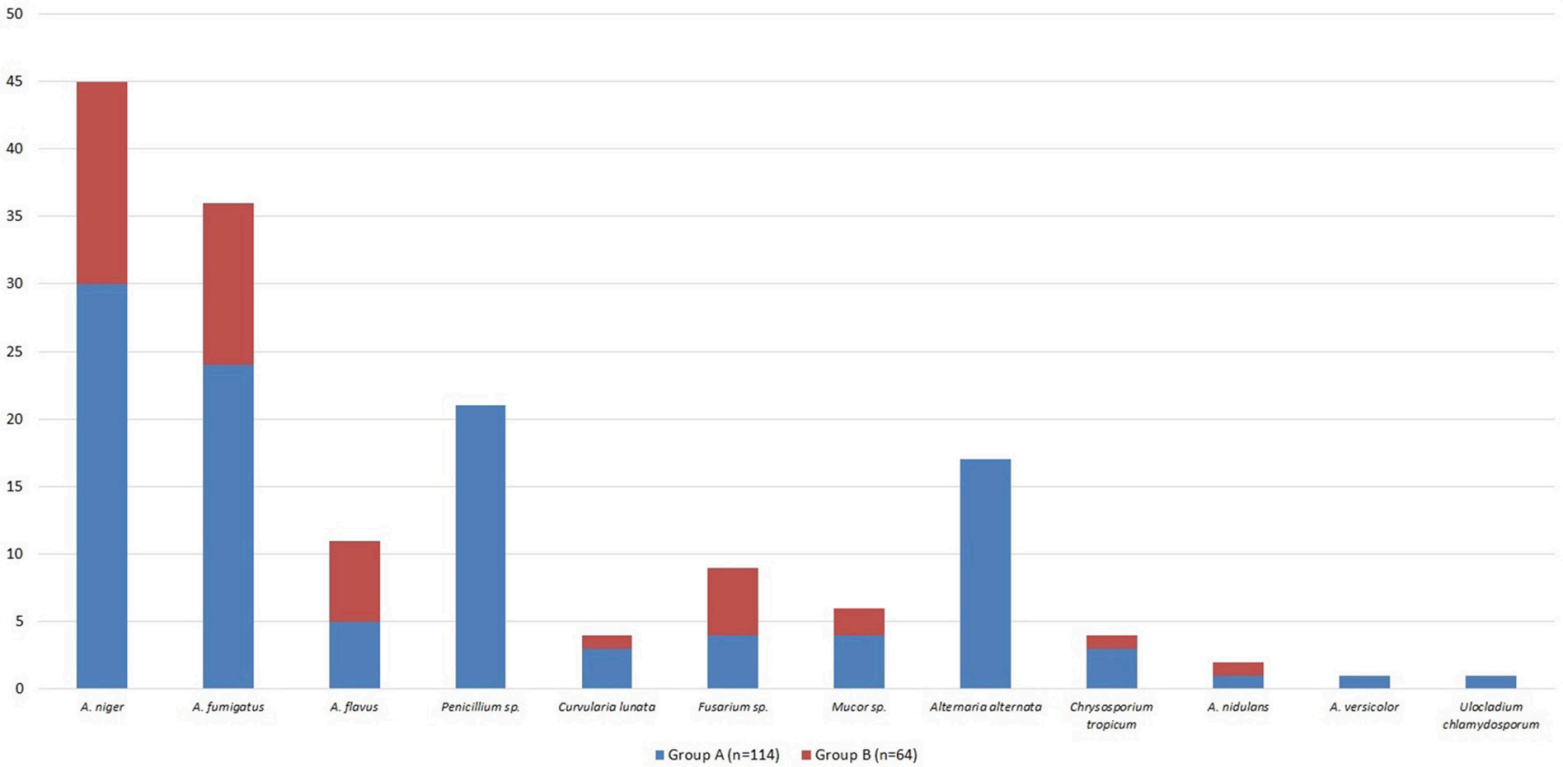
Fungal biodiversity in environment of patients from group A and B.

**Figure 6:**
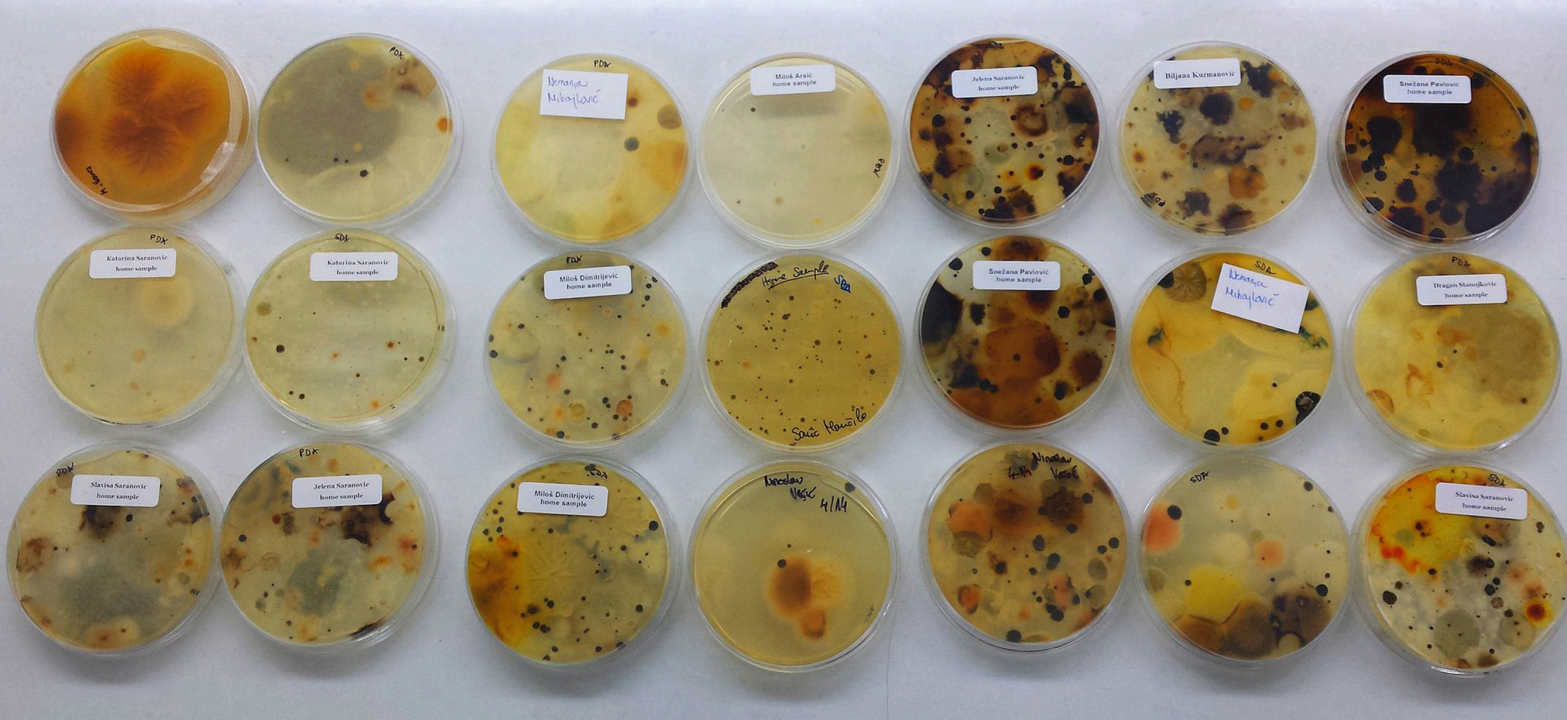
Variety of fungi isolated from air-samples sampled from bedrooms of patients with ABPA+AFRS.

Out of all fungal isolates (*n* = 178) from the air-samples obtained from the homes of patients with ABPA (one air sample per patient), 114 isolates were from the air-samples of group A, while 64 were isolated from air-samples of group B (Figure 5). The most predominant species in group A was *A. fumigatus* and *A. niger* isolated from 47% air-samples, followed by *Penicillium* sp. isolated from 18% air-samples and *Alternaria alternata* isolated from 15% air-samples (Figure 5). Macroscopic picture of fungal diversity in air-samples is present (Figure 6).

## Discussion

Our prospective cohort study revealed that the presence of AFRS in patients with ABPA should not be missed: (i) AFRS was confirmed in 80% of patients with ABPA; (ii) sinonasal allergic mucin was seen in all ABPA+AFRS patients and fungal hyphae in 60%; (iii) ABPA+AFRS patients had more often complicated CRS with NP (*p* < 0.001) and more severe forms of CRS with chronic durations of more than 10 years (*p* < 0.001), comparing to patients without AFRS; (iv) culture of sinonasal aspirate revealed fungal presence in 97% patients with ABPA+AFRS, while there was no positive fungal growth in the sinonasal aspirates of patients without AFRS; (v) patients with ABPA+AFRS had more common positive SPT for *A. fumigatus* (*p* = 0.037), while patients without AFRS had more common positive SPT for *Alternaria alternata* and *Penicillium notatum* (*p* = 0.04 and *p* = 0.032, respectively); (vi) 80% patients with ABPA and AFRS were classified as *Aspergillus* induced AFRS, as *Aspergillus* sp. was isolated from sinonasal aspirates of these patients (67% of all ABPA patients); (vii) larger number of fungi was isolated from the air-samples obtained from the homes of patients with ABPA+AFRS than from the homes of the patients without AFRS and the most predominant isolated species were *A. fumigatus* and *A. niger* found in almost 50% of the air-samples.

The prevalence of fungal airway's diseases, especially ABPA and AFRS, has been increasing every year last decades (Buzina et al., 2003; Huffnagle and Noverr, 2013). *Aspergillus* has ability to act as an antigen and to invade both, the lower and upper parts of respiratory tract, due to similarities between AFRS and ABPA (Mukherjee et al., 2015).

Although many studies tried to reveal the pathophysiology of ABPA and AFRS, it is still unclear. In patients with ABPA and AFRS, structural abnormalities in the airway epithelium, presence of sinonasal mucin and inappropriate clearance allows fungal growth (Chaudhary and Marr, 2011; Rick et al., 2016; Zhang et al., 2017). The presence of fungi increased neutrophils and eosinophils and IgE Ab (total and *Aspergillus*-specific) in these patients, causing chronic inflammation (Woolnough et al., 2015; Kumamoto, 2016). Formation of NP and bronchiectasis in AFRS and ABPA patients is consequence of chronic inflammation (Agarwal et al., 2017). Although ABPA and AFRS have many histopathological similarities, coexistence of both these clinical entities has not been reported often. Shah et al. revealed concomitant occurrence of ABPA and AFRS in only 7 of 95 patients by postoperative confirmation, while 38 had associated nasal symptoms and additional five asymptomatic patients had radiological evidence of sinusitis (Shah et al., 2001). Unfortunately, only nine patients signed written consent to undergoing surgical procedure for diagnosis. In other studies, ABPA with concurrent AFRS has also been rarely reported (Travis et al., 1991; Schubert and Goetz, 1998). However, in the present study, AFRS was confirmed in 80% ABPA patients, while *Aspergillus* induced AFRS was confirmed in 67% ABPA patients, by radiological and microbiological evidence. One reason for the high co-occurrence of ABPA and AFRS could be that all study patients were referred to an ENT-division due to sinonasal discharge. If all ABPA-patients and the ones without ENT-symptoms were screened, the rate would probably be lower. On the other side, the divergence in results could be explained by differences in the methodology used for fungal detection, isolation and identification. In the present study, we used new methodology for sampling and processing of sinonasal mucin and extraction of fungi from tick allergic mucin (Figure 1). In addition, this study was designed as prospective cohort study aimed to reveal the number of AFRS patients within ABPA group and relationship between clinical findings of these groups, in contrast to other studies that mostly were retrospective or review studies. In addition, in studies such as the one published by Shah et al. AFRS could not be ruled out in patients with sinusitis because some refused to undergo surgery, that is required for establishing diagnosis (Shah et al., 2001). It is supposed that the frequency of AFRS among patients with ABPA could be higher.

Tenacious secretions in bronchi of patients with asthma provide favorable environment for the fungal growth and subsequent release of antigenic material, which explain occurrence of ABPA in asthmatic patients (Woolnough et al., 2015; Rick et al., 2016). Similar chain of events may be responsible for AFRS onset (Woolnough et al., 2015). On the other side, the scenario could be reversed; the changes in the bronchial or sinus mucous and the secretory immune system exist in patients who develop ABPA and AFRS, favoring growth of fungi (Woolnough et al., 2015; Agarwal et al., 2017). In ABPA fungal diversity is increased, patients usually have poorly controlled asthma, recurrent pulmonary infection and bronchiectasis (Wieringa et al., 2001; Chishimba et al., 2015; Ryan and Clark, 2015). In the study of Chishimba et al. the relative abundance of *Aspergillus* increased approximately 15-fold in severe asthmatics compared to mild asthmatics (Chishimba et al., 2015). In our study, 77% patients with ABPA had only asthma, while 23% had asthma and bronchiectasis. Chest CT showed transient pulmonary infiltrates and central bronchiectasis in 23% patients. All patients have raised IgE Ab, while 72% had positive SPT for *A. fumigatus*. In the present study, 83% patients with ABPA had CRS, almost 50% had complicated forms with recurrent NP and severe subjective rhinological symptoms. In all our patients with ABPA and AFRS the chest symptoms preceded the nasal symptoms, but nasal symptoms were dominant, while in the patients without AFRS, presence of bronchiectasis was more frequent compared to patients with AFRS. Rhinological score for estimation of subjective symptoms revealed that 51.3% ABPA patients had severe nasal symptoms. Presence of sinonasal and sputum plugs is increased in patients with ABPA and AFRS (McCarthy and Pepys, 1971). McCarthy and Pepys, in their review of 111 patients with ABPA, reported the passage of sputum plugs in 54% and nasal plugs in 10% cases, but further investigation to confirm the diagnosis of AFRS has not been done (McCarthy and Pepys, 1971). Schubert and Goetz also reported passage of nasal and chest casts in 75% of their 67 patients with AFRS (Schubert and Goetz, 1998). Patient's observation that presence of both sinonasal and sputum secretion is increased should alert the physician to the possibility of coexistent AFRS and ABPA (McCarthy and Pepys, 1971; Schubert and Goetz, 1998). Future studies examining paired upper and lower airway mycobiota in individuals with CRS and asthma are desirable.

Although *Aspergillus* is the most common fungi isolated from respiratory tract of patients with ABPA or AFRS, some previous studies reported that patients with concomitant ABPA and AFRS were attributed to *Curvularia* spp. and *Bipolaris* spp. (Travis et al., 1991; Schubert and Goetz, 1998). These discrepancies could be explained by the cross-reactivity of allergens derived from common airborne fungi that could also play an important role in ABPA and AFRS patients (Kale et al., 2015; Chaaban et al., 2016). *Aspergillus* was the most common isolate from sinonasal aspirate in the present study, isolated from sinonasal aspirate of 80% patients with AFRS, and this group was classified as *Aspergillus* induced AFRS. *A. fumigatus* was isolated in 50% cases, followed by *A. flavus* (17%), *A. niger* (13%), and *Penicillium* sp. (10%). Air-samples from the houses of patients with AFRS and ABPA revealed that *Aspergillus* is the most common isolate, so this could be related to the frequent presence of *Aspergillus* in sinonasal aspirate. Considering various results of different studies, geographical differences in environmental and clinical fungal agents exist (Travis et al., 1991; Schubert and Goetz, 1998; Barac et al., 2015). Avoidance of places with fungal overload in air may help patients with ABPA or AFRS in the prevention of possible sharing of airborne fungal epitopes.

The drawback of the present study is that the molecular diagnosis of fungal isolates was not done, and consequently genotyping results were not available that should confirm if the same fungal strains were isolated form the patient's upper respiratory tract and air-samples. Air-sample fungal isolates were reported only as percentages, because the present study was focused on the clinical evaluation of diseases. The mycological study is started as continuation of the present clinical study, which is focused on a comparison of the strains isolated from sinonasal aspirates of the present cohort and air samples.

In conclusion, this first prospective study suggests that the co-occurrence of ABPA and AFRS is more common than previously expected. The pathogenesis of ABPA and AFRS share several common features, and AFRS can be considered as the upper airway counterpart of ABPA. Although these diseases are often treated by two different specialities, it seems that both are very much connected. This study confirms that the most common cause of these airway diseases is *Aspergillus*, and there are sufficient arguments to categorize this as united airway *Aspergillus* disease. Because *Aspergillus*-related upper and lower respiratory tract allergic diseases are inseparably connected, treating allergic *Aspergillus* lung diseases means also treating the nose, while treating patients with allergic *Aspergillus* sinonasal diseases has to be associated with a proper lung function evaluation, since the sinonasal and broncho-pulmonary systems should always be considered as a unique entity. Future studies should clarify the mechanisms by which *Aspergillus* turn from “normal flora” into the trigger of immunological reactions, resulting in ABPA or AFRS as well as to find new approaches for its' diagnosis and treatment.

## Author contributions

AB, SR and VT made substantial contributions to conception and design, and acquisition of data. PS and DO did a collection of data and statistical analyses. AP and LJ did an interpretation of data and final review of the paper. All authors contributed in writing the paper.

### Conflict of interest statement

The authors declare that the research was conducted in the absence of any commercial or financial relationships that could be construed as a potential conflict of interest.
